# Molecular profiling and antimicrobial potential of endophytic *Gliomastix polychroma* CLB32 inhabiting *Combretum latifolium* Blume

**DOI:** 10.1080/21501203.2015.1113207

**Published:** 2015-11-25

**Authors:** H.C. Yashavantha Rao, Syed Baker, Devaraju Rakshith, Sreedharamurthy Satish

**Affiliations:** a Microbial Drugs Laboratory, Department of Studies in Microbiology, Manasagangotri, University of Mysore, Mysore570 006, Karnataka, India; b Department of Plant Pathology, University of Georgia, Athens, GA30602, USA

**Keywords:** endophytic fungi, multidrug resistance, antimicrobial metabolites, TLC-bioautography

## Abstract

Fungal endophytes as a source of bioactive metabolites have led to the development of pharmaceutical products finding new applications. In a survey of endophytic fungal biodiversity, an antimicrobial endophytic strain CLB32 was isolated from the leaf of *Combretum latifolium* Blume (Combretaceae) from the Western Ghats of Southern India. CLB32 was then identified as *Gliomastix polychroma* (KR704576) by morphological and phylogenetic analysis based on internal transcribed spacer (ITS) nuclear rDNA and intervening 5.8S rRNA gene. CLB32 here constituted the first report on incidence of endophytic fungi from *C. latifolium* Blume. Ethyl acetate fraction of strain CLB32 was evaluated for antimicrobial activity by disc diffusion assay. Secondary metabolites produced effectively inhibited methicillin-resistant *Staphylococcus aureus* (18.33 ± 0.33 mm), *Pseudomonas aeruginosa* (14.66 ± 0.33 mm) and *Candida albicans* (14.00 ± 0.57 mm). Biosynthesis of these antimicrobial compounds was detected by analytical TLC-bioautography method as depicted by zone of inhibition on intensive the band. These findings suggest that *G. polychroma* CLB32, as a producer of natural antimicrobial drugs, could help to combat against multidrug-resistant infections and also provide baseline information for industrial applications.

## 1. Introduction

Emergence of multidrug-resistant (MDR) microorganism infections has generated considerable attention in recent decades (Boucher et al. 2008; Cars et al. 2008). Antibiotic resistance is one of the greatest challenges facing modern medicine (Spellberg et al. 2008). Antibiotics that lose their effectiveness for treating human disease through antibiotic resistance must be replaced with new drugs (Hamad 2010). The massive increases in trade and human mobility brought about by globalization have enabled the rapid spread of infectious agents, including those that are drug-resistant. World Health Organization (WHO 2014) report on global surveillance of antimicrobial resistance reveals that antibiotic resistance is no longer a prediction for the future, it is happening right now, across the world and is putting the treatment of common infections in the community and hospitals at risk.

Biodiscovery from microbial resources helps in the exploration of microbial metabolic products to detect, identify and evaluate their potential for medicinal, agricultural and biotechnological operations (Krutboke 2010; Baker et al. 2014). Plants lack immune response to certain pathogens, but the endophytes that reside inside the plant tissue enhance the immune response of the plants to fight against invading pathogens (Melotto et al. 2008). Fungal endophytes are polyphyletic group of highly diverse fungi that are defined functionally by their occurrence within tissues of plants without causing any immediate overt effects (Rodriguez et al. 2009). This endophyte–plant interaction induces the production of novel antimicrobial agents and endophyte–endophyte interactions within plants also have the potential to produce novel antimicrobial agents (Bandara et al. 2006; Nutzmann et al. 2011). In recent decades, endophytic fungi from plants have been widely accepted as major sources of drugs. A large number of bioactive compounds with new structures are continuously being isolated from endophytes (Strobel et al. 2004). They are strongly considered as largely unexploited metabolic resources. Therefore, new antimicrobial metabolites continue to be identified from fungal endophyte source.

*Combretum latifolium* Blume (Combretaceae) is a large climbing shrub that has great ethnomedicinal values (Shrisha et al. 2011). The stem and bark of this shrub are used as insecticides (Suthari et al. 2014). Leaf juice is used in the treatment for dysentery and goitre (Debnath et al. 2014). In view of this, *C. latifolium* Blume is selected to explore hidden potential endophytic fungi that can inhibit the growth of methicillin-resistant *Staphylococcus aureus* (MRSA) and other human pathogens.

## 2. Materials and methods

### 2.1. Study site location and source of endophytic fungi

Pushpagiri Sanctuary (12°35′N 75°40′E, elevation 1748 m) is located in the Western Ghats of Kodagu (Coorg), Karnataka, which is a part of Southern India. It is covered with thick evergreen forests and shoal grassland habitat. It is one of the eight ‘hottest hotspots’ of biological diversity in the world (Myres et al. 2000). *C. latifolium* Blume from this region was selected for the study.

### 2.2. Collection of samples

Specimens of *C. latifolium* Blume were carefully collected. In order to secure the endophytic nature of the isolates, the cut ends of bark and root specimens were sealed with wax. All the specimen samples were brought to the laboratory in an icebox and stored at 4°C. Each sample was used for the isolation of endophytic fungi within 24 h of collection.

### 2.3. Isolation of endophytic fungi

Isolation of endophytic fungi was carried out according the method described by Wang et al. (2012) with some modifications. Each sample tissue was washed under running water for 15 min and dried at room temperature prior to surface-sterilization. All the slight, visibly damaged material was excluded. To remove the epiphytic microorganisms, samples were rinsed with 70% ethanol for 2 min, surface-sterilized by sodium hypochlorite (4%) for 5 min and rinsed with 70% ethanol for 30 s. Then the sample tissues were rinsed with sterile double distilled water and kept for surface drying in sterile conditions. To confirm the success of the surface disinfection process, aliquots of the sterile distilled water from the final rinse were inoculated on the isolation media plates. The plant tissue samples were cut into small segments (5 mm size) and placed on water agar plates (distilled water, 1.5% agar) amended with chloramphenicol (250 ppm) and incubated at 30°C for 3–4 days to few weeks, until the growth initiated. The hyphal tips that emerged from the plant tissues were picked and maintained on PDA plates for further studies.

### 2.4. Molecular profiling of endophytic strain CLB32

#### 2.4.1. Genomic DNA extraction

*G. polychroma* was cultured in PDB for 7 days at 30°C, and the mycelium was harvested by vacuum filtration. The chilled mycelia were ground with pestle and mortar under liquid nitrogen, transferred into the micro centrifuge tube with 1 ml of 2× CTAB extraction buffer and incubated at 65°C for 30 min with gentle swirling. After centrifugation, the aqueous phase of the mixture containing total DNA was extracted with an equal volume of phenol:chloroform:isoamyl alcohol (25:24:1). Residual phenol was removed with the addition of chloroform:isoamyl alcohol (24:1) twice. Two volumes of ethanol and 0.1 volume of 3 M sodium acetate were added to the aqueous phase of DNA for precipitation and then incubated at −20°C overnight (Kim et al. 2010). The DNA pellet was washed with 70% ethanol twice, and suspended in 15 µl of TE buffer. Polymerase chain reaction (PCR) amplification was carried according to the protocol of Bhagat et al. (2012) using ITS1 (5ʹ TCCGTAGGTGAACCTGCGG 3ʹ) and ITS4 (5ʹ TCCTCCGCTTATTGATATGC 3ʹ) universal primers (White et al. 1990).

#### 2.4.2. Taxon sampling and phylogenetic affiliation

Internal transcribed spacer (ITS) sequence data of strain *G. polychroma* CLB32 obtained was annotated using Geneious 6.1.6 (Biomatters, 2013, Auckland, New Zealand) software and submitted to the National Centre for Biotechnology Information (NCBI) GenBank. ITS rDNA sequences with maximum identity to that of strain CLB32 were retrieved from NCBI nucleotide database using Basic Local Alignment Search Tool (BLAST). ITS sequences were filter-searched, and the closest sequences were selected for the phylogenetic analysis. The multiple sequence alignments were performed using CLUSTALW software utilizing default settings, and dendrograms were generated by MEGA 4.0 software with a bootstrap consensus of 1000 replicates (Padhi and Tayung 2013).

### 2.5. Evaluation of antimicrobial activity

Antimicrobial susceptibility testing of ethyl acetate extract was carried out by the disc diffusion assay (Sadrati et al. 2013). Sterile discs (6 mm dia) impregnated with 20 μl of ethyl acetate extract were dried in laminar hood and placed on the surface of the media seeded with test pathogens in Petri plates. One negative control disc impregnated with only 20 μl of ethyl acetate was also placed for each test organism with Gentamicin as a positive control. The plates were incubated at 37 ± 2°C and diameter of the zone of inhibition was recorded.

### 2.6. Thin-layer chromatography-bioautography

Ten microlitres of ethyl acetate extract was spotted on TLC silica gel plates (TLC, Alugram® SIL G/UV_254_; Machereye-Nagel, Duren, Germany) in petroleum ether/ethyl acetate (1:2) optimized solvent system. The developed TLC plates were observed under visible light and UV light at 254 and 365 nm, respectively. TLC plates were then air-dried for complete removal of the solvent traces and UV sterilized for 30 min. TLC plates were then encased in sterile Petri plates overlaid with Brain heart infusion medium containing 0.65% agar incorporated with 1 mg ml^−1^ concentration of 2,3,5-triphenyl tetrazolium chloride (TTC) (Sigma-Aldrich) inoculated with 1% standardized MRSA inocula. After 8 h of diffusion at 8°C, the plates were incubated for 24 h at 37°C. The areas of inhibition zones on the active spot were recorded (Valgas et al. 2007).

## 3. Results and discussion

### 3.1. Isolation of endophytic fungi

**Figure 1.**
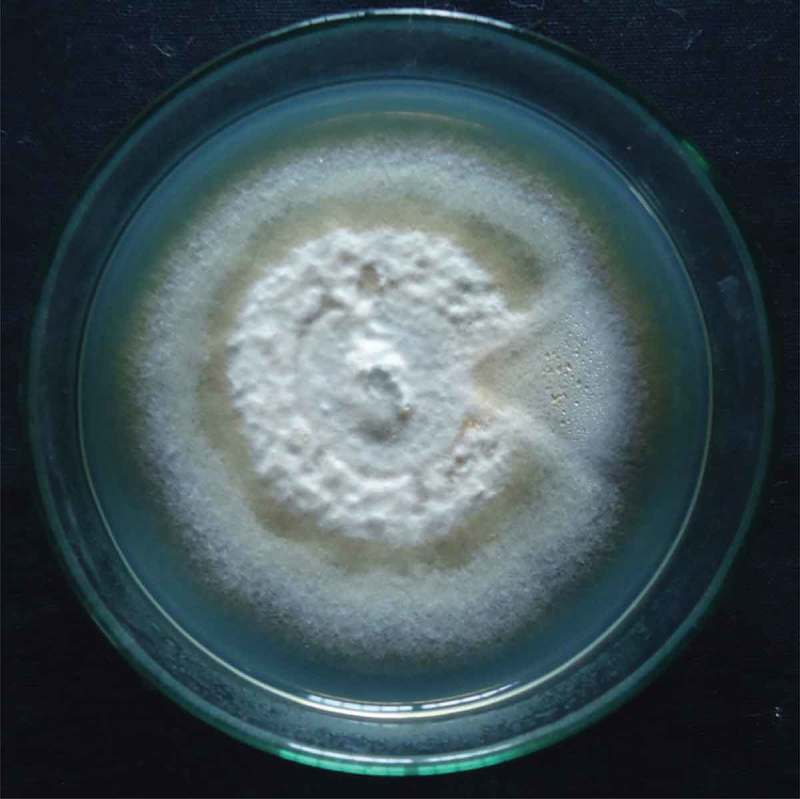
Colony morphology of *Gliomastix polychroma* CLB32 on potato dextrose agar after 10 days.

It has becoming evidence that all higher plants host has one or more untapped endophytic microorganisms (Strobel 2003). The results obtained in the present investigation attribute considerable interest towards endophytic research. In this study, an endophytic fungus, *G. polychroma* CLB32, was isolated from the asymptomatic bark tissue of *C. latifolium* Blume. In addition, media Petri plates spread with the final rinse of sterile water showed no microbial growth even after 10 days of incubation at 28°C. This indicates that the surface sterilization method was effective in killing the epiphytic microorganisms. Thus, subsequent isolates can be considered as true endophytic fungi (Barnett and Hunter 1956). Earlier studies also define the successful surface sterilization process for the isolation of endophytes with no contamination of epiphytes (Kusari et al. 2009; Lin et al. 2010). The isolated endophytic fungus was then identified as *Gliomastix* sp. on the basis of morphological characteristics of growth pattern, colour of colony, surface texture, hyphae, margin and characteristics of the spores (Figure 1). Further to identify the strain at species level molecular analysis has been carried out. This study constitutes the first report on incidence of endophytic fungus inhabiting *C. latifolium* Blume.

### 3.2. Taxon sampling and phylogenetic affiliation

**Figure 2.**
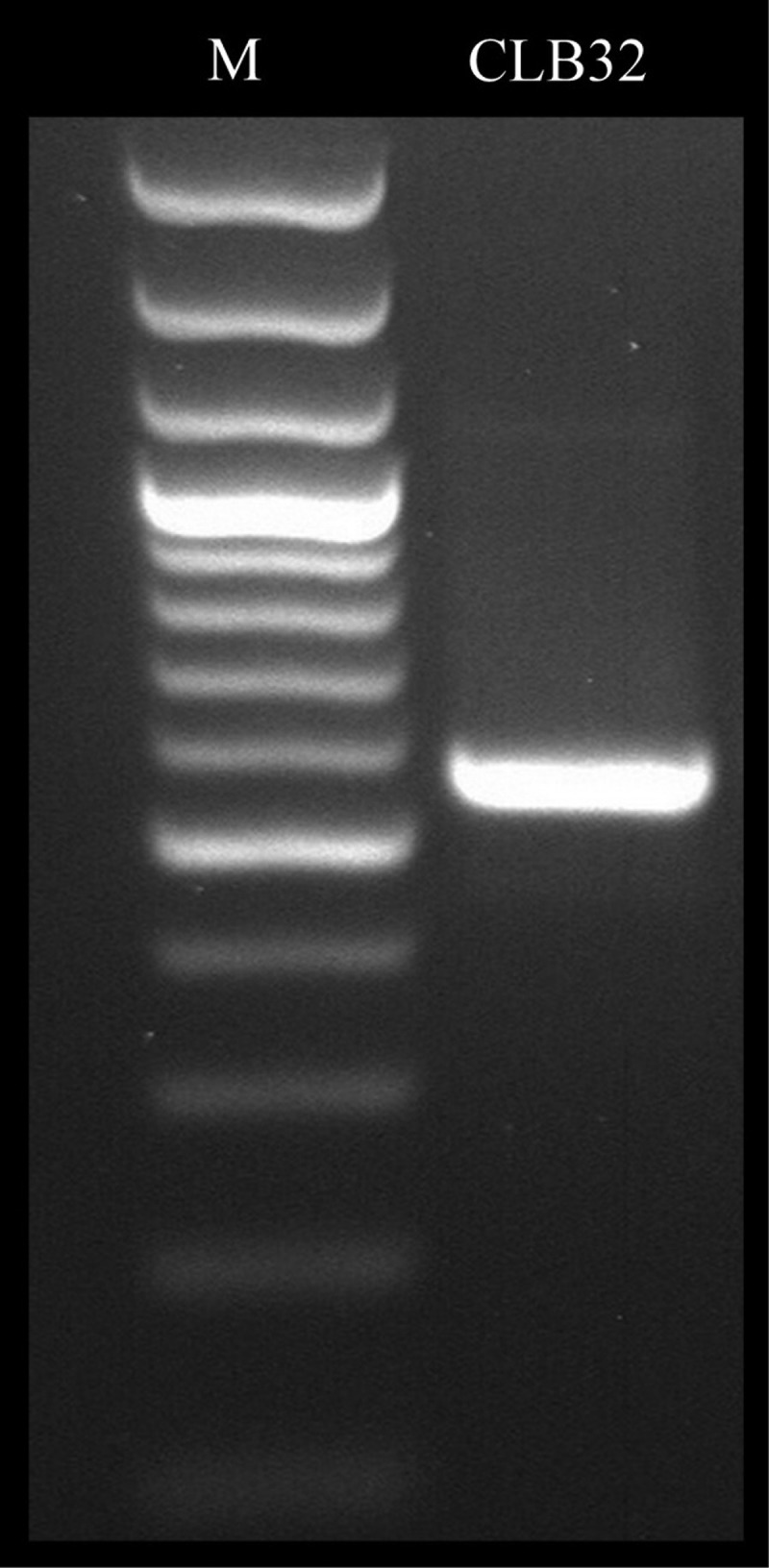
Polymerase chain reaction (PCR) amplification of *Gliomastix polychroma* CLB32 rDNA by internal transcribed spacer (ITS1 and ITS4) universal primers.

Note: Lane: M = 100 bp DNA ladder, CLB32 = ~580 bp amplicon representing ITS region of rDNA.

**Figure 3.**
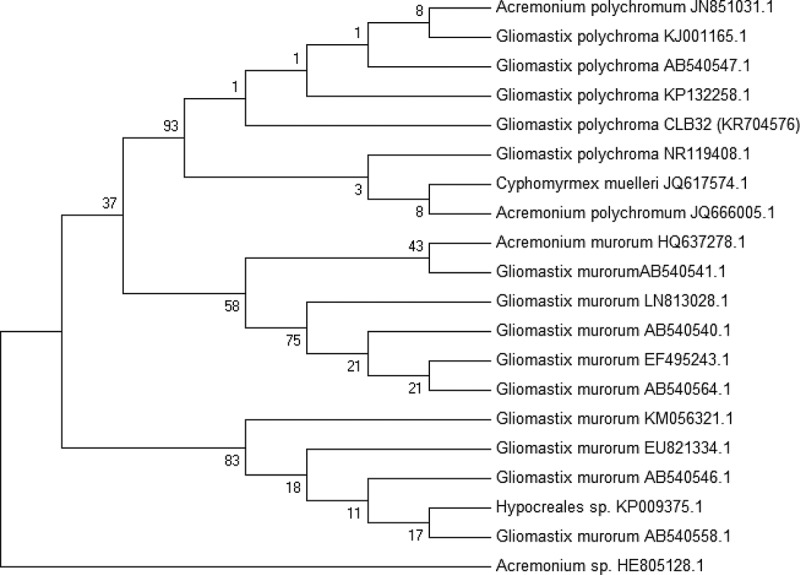
Phylogenetic tree derived from NJ analysis showing the evolutionary relationship of *Gliomastrix polychroma* CLB32 with its closest BLAST hits. Bootstrap values (1000 replications) based on multiple sequence alignment using the MEGA-5 software.

Strain CLB32 was identified using both morphological characters and phylogenetic data for precise identification. Phylogenetic affiliation was carried out based on ITS region of rDNA and intervening 5.8S rRNA gene (Figure 2). The amplified ITS region of rDNA was sequenced and aligned with ITS sequences of the different strains retrieved from NCBI databases using CLUSTALW (Thompson et al. 1994). Alignment with those from NCBI database resulted in several closely related sequences. Corresponding neighbour joining (NJ) tree showed clearly that strain CLB32 fell into the group of *Gliomastix polychroma* with strong support (Figure 3). ITS rDNA sequence profiling is one of the cost-effective tools that enable the researchers to identify the complex microbial communities in species level at exceptional depth and resolution (Rastogi and Sani 2011). The number of previously phylogenetically unstudied fungi is large (Summerbell et al. 2011); however, morphological identification has long been the basis and tradition, which is, beyond doubt, of great importance (Wang et al. 2014). Thus combined with morphology and phylogenetic data, strain CLB32 was defined as *Gliomastix polychroma* ultimately. The ITS sequence data of this fungus is deposited in GenBank under the accession no. KR704576.

### 3.3. Antimicrobial susceptibility testing

**Figure 4.**
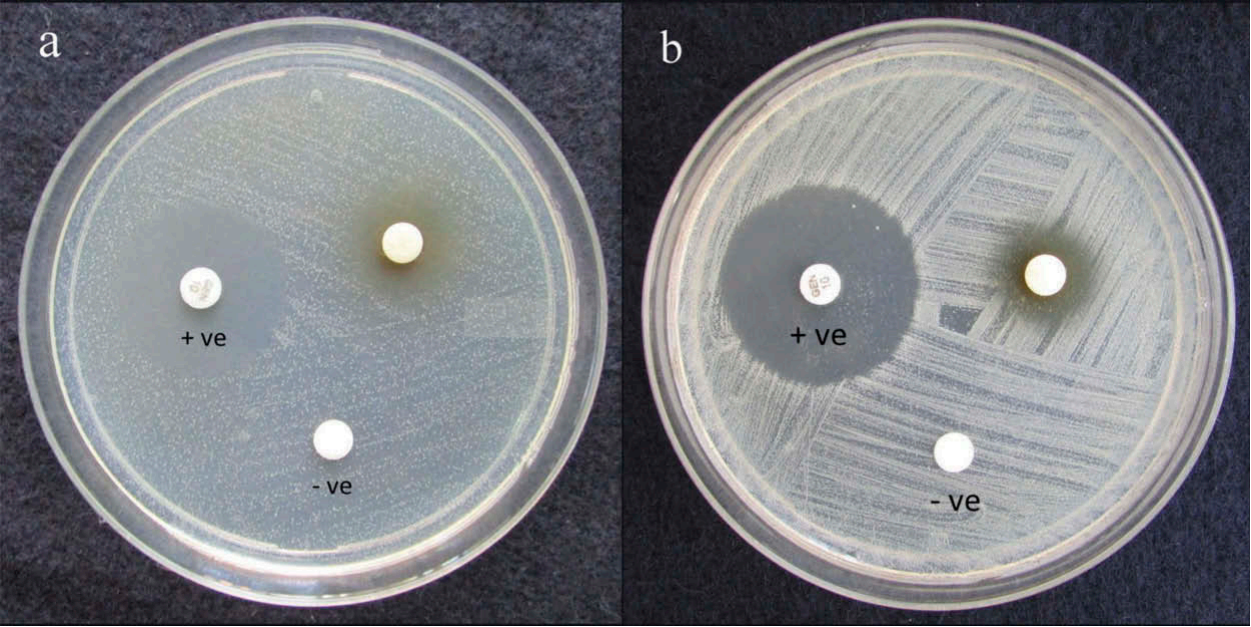
Antimicrobial activity of ethyl acetate extract of endophytic *Gliomastix polychroma* CLB32 against (a) MRSA, (b) *Salmonella typhi*.

Note: +ve = Gentamicin as positive control; –ve = Ethyl acetate as negative control.

Antimicrobial potential of ethyl acetate extract of CBL32 was analysed by disc diffusion assay (Figure 4). Ethyl acetate has been used for the extraction of secondary metabolites as most of secondary metabolites are miscible with ethyl acetate with strong literature support (Jumpathong et al. 2011; Rao and Satish 2015). The results obtained were validated with standard antibiotics, namely gentamicin for antibacterial activity and nystatin for antifungal activity. During antimicrobial susceptibility test, strain CBL32 displayed strong antimicrobial activity, where significant activity was observed against MRSA (18.33 ± 0.33 mm), followed by *E. coli* (17.66 ± 0.33 mm) and *Pseudomonas aeruginosa* (16.33 ± 0.66 mm). The strain also showed antifungal activity against *Candida albicans* and *Microsporum canis* (Table 1). The ability to suppress the growth of both bacterial and fungal pathogens implies that metabolites have a broad spectrum of antimicrobial activity (Rao et al. 2015). This fungus, as an endophyte, may be involved in protecting the host against invading pathogens, and this could have led to the ability of this endophyte to biosynthesize some phytochemicals originally associated with the host (Stierle et al. 1993, Wang et al. 2012). *Streptomyces longisporoflavus* as endophyte was isolated from *L. ciliata* Benth collected from Western Ghats, India, which exhibited anti-diabetic activity (Akshatha et al. 2014). Antimicrobial activity of endophytic fungi inhabiting eight medicinal plant species sampled in different locations from the Western Ghats of India was recorded (Raviraja et al. 2006). Therefore, the present work expands our understanding of potential hidden endophytic fungi underexplored area are the promising source for novel antimicrobial agents. Biodiscovery of antimicrobial producing endophytic fungi associated with *C. latifolium* Blume is valuable for industrial interest and for basic research (see supplemental data).

**Table 1.** Determination of antimicrobial activity of ethyl acetate extract of *Gliomastix polychroma* CLB32 (100 μg/disc) by disc diffusion assay.

Test pathogens	Zone of inhibition (mm)	Standard (mm)
MRSA	18.33 ± 0.33^a^	24.00 ± 0.00^b^ (G)
*Staphylococcus epidermidis*	14.66 ± 0.33^c^	24.66 ± 0.33^b^ (G)
*Salmonella typhi*	15.33 ± 0.33^bc^	26.33 ± 0.00^a^ (G)
*Pseudomonas aeruginosa*	16.33 ± 0.66^abc^	24.66 ± 0.33^b^ (G)
*Escherichia coli*	17.66 ± 0.33^ab^	26.00 ± 0.00^a^ (G)
*Candida albicans*	14.00 ± 0.57^c^	19.66 ± 0.33^c^ (N)
*Microsporum canis*	14.33 ± 0.66^c^	19.00 ± 0.00^c^ (N)

Note: Values represent the diameter of the zone of inhibition. Data are means from three replicates ± SE and those with the same superscript letters in the appropriate columns are significantly different (ANOVA, Tukey’s HSD at *p* < 0.05). G = Gentamicin (100 μg/disc), N = Nystatin (100 μg/disc).

### 3.4. Thin layer chromatography-bioautography

Chromatographic profile of ethyl acetate extract of CLB32 signalled the presence of an intense band under 254 and 365 nm, which might be due to the increased production of secondary metabolites. In the bioautography assay, a clear zone of inhibition was observed on the intense band against the red background where the medium was preinoculated with MRSA and TTC agent. This confirmed that the presence of antimicrobial compound in the ethyl acetate extract, which was active against MRSA strain. Detection of antimicrobial compound by TLC-bioautography is one of the economical, simplest and reproducible methods for drug discovery from natural products (Hota 2010; Patra et al. 2012). The antimicrobial activity of ethyl acetate extract of *Phomopsis* sp. FPSP-25 was determined using the same approach (Rakshith et al. 2013). Further chemical investigation is needed to characterize the metabolite to combat against human pathogens and MDR microorganisms for antibacterial activity.

## 4. Conclusion

This work suggests that the screening of potential endophytic actinomycetes from an underexplored area can give a new facelift for antimicrobial research and drug development. Effective inhibition of MDR microorganisms by this fungus implies that this strain could be a reliable source for industrially and pharmaceutically important bioactive compounds. This work is the first report on the incidence of endophytic fungi inhabiting *C. latifolium* Blume.

## Disclosure statement

No potential conflict of interest was reported by the authors.
